# Data-driven analysis and new findings on the loss of tail rotor effectiveness in helicopter accidents

**DOI:** 10.1038/s41598-022-06647-0

**Published:** 2022-02-16

**Authors:** Joseph Homer Saleh, Zhaoyi Xu, Anca Ioana Guvir, Arega Margousian, Weiqing Zhang, Martin Ma

**Affiliations:** School of Aerospace Engineering, Georgia Institute of Technology, Atlanta, GA USA

**Keywords:** Engineering, Aerospace engineering

## Abstract

Loss of tail rotor effectiveness (LTE) is an unstable dynamic phenomenon that affects single-rotor helicopters and frequently leads to accidents. LTE accidents recur with troubling regularity and show no sign of abatement. This work uncovers new data-driven findings pertaining to LTE and risk factors. First, a scorecard is developed covering a broad range of results to better understand LTE accidents. Second, the risk of LTE is derived for current helicopters. Third, a Deep Learning model is developed that captures the dependence between LTE risk and helicopter features. A danger zone is discovered in the design space for short tail rotor arm and high tail rotor RPM. The results challenge the prevailing narrative of LTE accidents as mere pilot errors and demonstrate an intrinsic propensity to these accidents is embedded in part in the helicopter design. The findings open the door to new, more effective safety interventions for LTE accident prevention.

## Introduction

Loss of tail rotor effectiveness (LTE), also known as unanticipated yaw, is a distinctive dynamic phenomenon that affects single-rotor helicopters and frequently leads to loss of control resulting in accidents and casualties. In the United States, about 5–10 helicopter crashes occur every year due to LTE. A low-speed phenomenon, LTE depends on several factors, in particular the direction and strength of the wind relative to the helicopter, and it manifests itself in a seemingly un-commanded yaw motion generally in the opposite direction of the rotation of the main rotor blades. The helicopter appears to initiate a yaw motion, un-commanded and against the will of the pilot. The rotation is not stable, and if not vigorously corrected, the spin rate can increase dramatically until a crash occurs. LTE can be startling to pilots; its onset is sudden and its growth surprisingly energetic.

Although LTE manifests itself in a similar fashion to a tail rotor malfunction or loss of anti-torque system, the pedals feel unresponsive in both cases, it is not related to any physical breakdown in the system. The tail rotor remains fully operational during this phenomenon and its effectiveness undiminished. The misdiagnosis of an LTE as a tail rotor failure can induce pilots to make inadequate use of the pedal (instead of applying and maintaining full opposite pedal), which further aggravates the situation and contributes to an accident. The supplementary material includes two short videos (Videos 1, 2) that capture the onset of LTE and subsequent uncontrollable spin and crash. The videos are informative and sobering to watch, and they provide a better appreciation of this distinctively dangerous phenomenon in single rotor helicopters.

Interestingly, despite its importance for helicopter operation and safety, LTE is a relatively recent discovery, its identification dating back to the 1980s. We highlight next four notable milestones in the history of LTE. At the beginning, “in the early 1980s, the US Army was experiencing a curious phenomenon that was affecting their helicopters. They were [occasionally] losing two Bell OH-58 helicopters per month to un-commanded and uncontrollable spin accidents”^1^. The Army convened an inquiry board to investigate this unsettling problem. After a faulty start in which it was erroneously concluded that tail rotor was stalled during an LTE, the Army established a Joint Special Study Group (JSSG) in collaboration with Bell Helicopters Textron to test, analyze, and determine corrective actions for this phenomenon^2,3^. The group made a number of discoveries, for example that “certain relative wind directions are more likely to cause tail rotor thrust variations than others, [and that] these relative wind directions form the LTE conducive environment”. More importantly, the group found that the tail rotor was not stalled during this phenomenon, and this led to the recommendation of a new recovery technique, which called for full opposite pedal and forward cyclic. These findings remain the bedrock of today’s understanding and handling of LTE.

Around the same time, the National Transportation Safety Board (NTSB) investigated several instances of LTE accidents with the Bell 206, the civilian version of the OH-58. The Board issued recommendations to the Federal Aviation Administration (FAA), in 1984 and again in 1994, to include information on LTE in flight manuals:

“*The Safety Board has found no civilian helicopter flight manuals that contained information on LTE. […] The Safety Board considers proper documentation of this phenomenon in the flight manuals to be vital to the safe operation of [single rotor helicopters]. … The Board is concerned that civilian pilots unfamiliar with LTE may be vulnerable to [such] accidents*”^4^.

The NTSB went on to recommend that the FAA (1) issues a Flight Safety Notice that includes information about LTE, (2) encourages manufacturers to include in the operator’s handbook and flight manual a discussion of LTE and recovery techniques, and (3) amends the helicopter test standards to include references and questions about LTE^4^.

This prompting led to the third notable milestone in the history of LTE. In 1995, the FAA issued the Advisory Circular AC-90-95 in which the LTE phenomenon was discussed, the conditions under which it can develop explained, and the recommended recovery techniques laid out^5^. The circular contained essentially the same information the Army had presented about a decade earlier on LTE and the same recommended recovery technique. The FAA circular nonetheless carried significant weight since it was issued by a regulatory organization, which the NTSB is not, and it had significant reach within the rotorcraft community.

Some research was conducted and published on the topic as well^6–8^. Although not part of the four notable milestones, we briefly mention this body of literature. For example, Srinivas, Chopra, Haas, and McCool developed a helicopter tail rotor model for predicting thrust and yaw control effectiveness in low-speed flight regime and under different wind azimuth conditions^9^. Fletcher and Brown examined the aerodynamic interaction or wake coupling between the main rotor and tail rotor, and they assessed its implication for tail rotor thrust variation and helicopter directional control^10,11^. Cuzieux, Basset, and Desopper reviewed the three aerodynamic conditions that can lead to LTE, namely main rotor disk vortex interference with the tail rotor, tail rotor vortex ring state, and weathercock stability^12^. The authors also reported on the modeling and integration of these conditions in their Helicopter Overall Simulation Code (HOST). Gasparovic, Kovacs, and Fozo^13^ investigated the vortex ring state (VRS) in the popular medium-lift Russian helicopter Mi-17, which also experiences frequent LTE accidents. “Statistics from the territory of the former Soviet Union suggests that such accidents are not rare”^13^. The authors identified the impact of the VRS on the tail rotor thrust curve, and the conditions under which they lead to LTE.

The last notable milestone in our brief history of LTE occurred in 2017. The NTSB issued a Safety Alert (SA-062) in which it discussed again LTE and urged pilots to be alert to this problem and reminded them how to deal with it^14^. The Safety Alert referenced 55 recent NTSB investigations in which LTE was a contributing factor to the accident. This was a recognition of the persistence of LTE accidents, and that the problem is still not under control almost four decades after it was first identified.

It is useful at this point to step back and reflect on the current status in the history of LTE. Two things are clear. First, the phenomenon has been identified, its aerodynamic basis reasonably well understood, and the rotorcraft community is attentive to this problem (including the NTSB, FAA, helicopter manufacturers, and pilot associations). Second, like the war on drugs, the “war” on LTE has been a failure, as these accidents recur with a troubling regularity and show no sign of abatement. Beyond their statistics, which will be examined shortly, the price of these accidents is continued loss of life and limb and millions of dollars in material damage. For more progress to be made toward reducing this type of accidents, it is important to ask new research questions about this phenomenon and to examine new venues for understanding and tackling it. We explore new research directions in this work and uncover novel data-driven findings pertaining to LTE.

This work makes three contributions. First, an LTE scorecard is developed, and it covers a broad range of descriptive statistics to help better understand the epidemiology of LTE accidents. The scorecard identifies, for example, the top 4 helicopter models that contribute to 80% of LTE accidents, and it displays the trend, geographic locations, and phase of flight when these accidents occurred. Second, after a discussion of measures of exposure, the risk (or incidence proportion) of LTE accident is provided for different helicopters. The results identify, for example, the models most prone to such accidents, and they show that the risk of LTE accident in helicopters with reciprocating engines is about twice as high as that in helicopters equipped with turboshaft engines. Third, a highly accurate Deep Neural Network model is developed, which captures the dependence between the incidence proportion of LTE accidents and 12 different helicopter features (discussed in the next section). The key finding is the existence of a danger zone in the feature space at the intersection of short tail rotor arm and high tail rotor RPM (risk factors). The results identify, for example, that the combination of short tail rotor arm and high tail rotor RPM is associated with a distinctively high risk of LTE, and that by increasing the main rotor RPM, the danger zone further expands along with the risk of LTE. Similarly, it was found that the combination of high main rotor tip speed and high tail rotor tip speed is associated with a higher risk of LTE, and that increasing the disk loading further expands the LTE danger zone.

Why should these findings matter? It is generally accepted in the rotorcraft community that 60–70% of all helicopter accidents are due to pilot errors, including LTE accidents. The present work challenges this myopic narrative in the context of LTE accidents and demonstrates that an intrinsic propensity to these accidents is embedded in part in the helicopter design configuration beyond merely pilot errors. This perspective opens the door to new, possibly more effective safety interventions for LTE accident prevention, as will be argued next.

## Results

The findings are twofold and reflect the two complementary approaches adopted in this work. The first set consists of statistical results pertaining to the type of accidents examined and presented in the form of an LTE scorecard, followed by a discussion of a measure of exposure and the derivation of an LTE risk score for different types of helicopters. The second set of results is based on a highly accurate LTE prediction model developed with the tools of deep learning (DL), which identifies LTE danger zones in the design space of helicopter configuration.

Publicly available data of civil helicopters from three sources were used in this work: (1) the Federal Aviation Administration (FAA) for registration records; (2) the National Transportation Safety Board (NTSB) for accident records; and (3) helicopter manufacturer manuals for technical information. The data includes all registered helicopters in the U.S. civil fleet between January 2005 and December 2015, excluding gyrocopters, home-built and experimental helicopters. Additionally, the type of helicopter known as NOTAR (for No TAil Rotor) were removed, and so were the data recorded in geographic regions within the domain of the FAA but not classified as States, such as Virgin Islands and Puerto Rico (for data quality considerations). The final dataset consists of 11,998 helicopters and 71 LTE accidents. For each helicopter, a dozen features were collected including the number of main rotor blades, the main rotor diameter and its RPM, the number and type of engines (reciprocating or turboshaft), the engine horsepower, the maximum takeoff weight of the helicopter, the number of tail rotor blade, the tail rotor diameter and its RPM, the tail rotor type (traditional bladed or Fenestron), and the tail rotor arm (the horizontal distance between the center of the main rotor to the center of the tail rotor). Other features such as chord lines or solidity factors for the main and tail rotor blades were not available and could not be included in the analysis.

### LTE accidents scorecard

First, we develop a scorecard of LTE accidents during the 2005–2015 time period covering different statistics of these accidents. While this constitutes a useful first step toward a better understanding of these accidents, the scorecard is only based on NTSB (numerator) data and does not include measures of exposure to enable inferential and comparative analysis.

**Figure 1:**
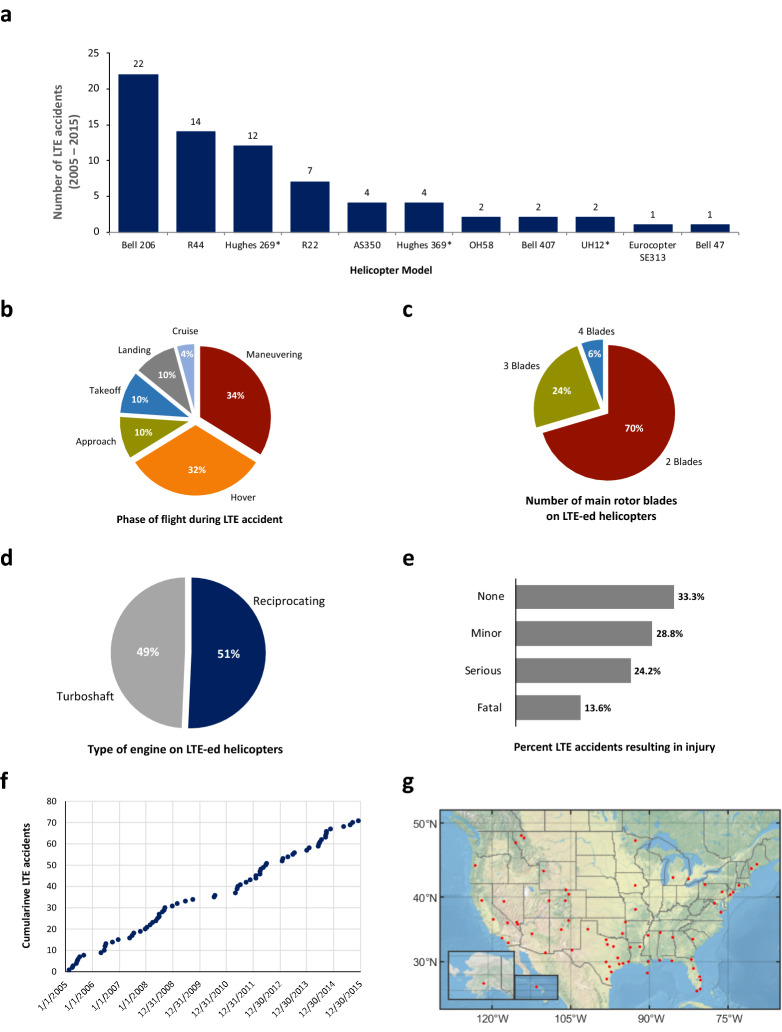
LTE accidents scorecard (2005–2015). Each panel presents a different aspect of these accidents (n = 71). (**a**) The number of LTE accidents by helicopter model. We have bundled the same helicopter models that have changed names or ownership, for example, Hughes 269 and Schweizer 269 are listed under “Hughes 269”. Similarly, the Hiller UH12 and the Hiller OH23 are listed under “Hiller UH12”. The two OH58 accidents are included here because they were flying under “public aircraft” (not military) for a police department and a Texas parks and wildlife department. The Hughes 369 is bundled with the OH-6 and MD500. (**b**) The phase of flight during which the LTE accident occurred. (**c**) The number of main rotor blades of the helicopters that experienced LTE accidents. (**d**) The type of engine of the helicopters that experienced LTE accidents. (**e**) The percent of LTE accidents that resulted in a given type of injury (only the most severe injury is counted in each accident, for example an accident that resulted in one fatality and one minor injury is only counted as a fatal LTE accident). (**f**) Cumulative LTE accident over 2005–2015. (**g**) Locations of LTE accidents in the U.S. over 2005–2015^15^.

The scorecard in Fig. 1 provides a broad set of results related to LTE accidents. For example, four helicopter models account for about 80% of all LTE accidents in the U.S. (Fig. 1a), namely the Bell 206, the Robinson R44 and R22, and the Hughes 269. The majority of LTE accidents occurred while the helicopter was maneuvering or hovering (Fig. 1b). There is a prevalence of helicopters with 2 main rotor blades in LTE accidents, in sharp contrast with 4-bladed ones (Fig. 1c). Both types of engines, reciprocating and turboshaft, are equally represented in these accidents (Fig. 1d), and about 40% result in fatal or serious injuries (Fig. 1e). The trend in LTE accidents is not abating, with 5 to 10 such crashes every year (Fig. 1f). The scorecard also shows the location of LTE accidents (Fig. 1g): along the Gulf Coast and Tornado Alley, around the Rocky Mountains, and on both East and West coasts.

The results in this scorecard are important, but they should be interpreted with care and not used beyond their expressive ability. For example, while the Bell 206 is the most prevalent family of helicopters in LTE accidents, it is also more represented in use in the U.S. fleet than the other models in Fig. 1a. As a result, it should not be concluded that the risk of LTE accident is highest for the Bell 206. Similarly, the results in Fig. 1d should not be interpreted to indicate that the risk of LTE accident is roughly equal for reciprocating and turboshaft engines since these concern numerator data only and do not account for measures of exposure. Helicopters with reciprocating engines are significantly more prevalent in the U.S. than ones with turboshaft engines, and this will shape the conclusions that can be deduced, as will be shown next.

### Measure of exposure

The preferred measure of exposure for this analysis is helicopter flight-hours and usage. Unfortunately, limited such data are collected in the U.S. except for a small voluntary survey of helicopter operators by the Federal Aviation Administration (FAA). In addition to the voluntary response bias in this small sampling exercise, the survey results are not usable in this study for two reasons: (1) the survey lumps different configurations of helicopters together, for example, all twin-engine turboshaft helicopters are lumped into a single category, and a single estimate of the flight hours per category of helicopter is provided. This is not appropriate since, for example, 2-bladed, 4-bladed, and 5-bladed helicopters can be found with twin engines and have different intensity of use. As a result, the estimate provided likely aggregates highly different (multi-modal) data points for which a small sample mean is an otiose statistic; (2) more importantly for the present study, since the focus is on investigating the impact of choices in the design space of helicopter configuration on the risk of LTE accidents, the absence of such information in the FAA survey estimates renders them unusable for our purposes. Several authors have commented on this state of affairs. For example, Fox (2005) noted that “the lack of flight-hour exposure data [is a major] roadblock to the helicopter industry. If we cannot measure risk, we cannot tell whether our improvement is an actual one or whether it made the problem worse”^16^. Knetch and Smith (2012) also acknowledged this limitation and noted that, “some readers will be disappointed and would prefer to see a study based on, say, accident per flight-hour, or per departure. Unfortunately, that kind of [exposure] data is not available”^17^. In the absence of such data and the subsequent inability to estimate LTE accident rates, we investigate what epidemiologists refer to as incidence proportion^18^. This is the number of LTE accidents experienced by a particular model/family of helicopters per the number of such helicopters registered as active in the U.S. with the FAA.

### Risk of LTE accidents

**Figure 2:**
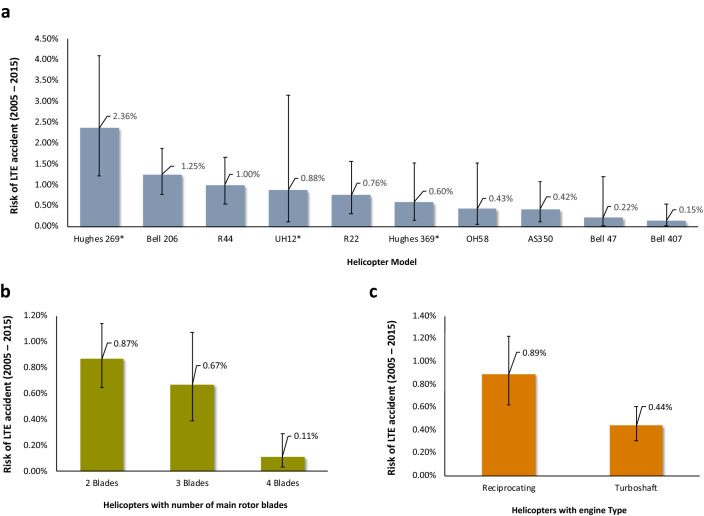
Risk of LTE accidents (2005–2015). Each panel presents different aspects of the risk of LTE accident (n = 71. Error bars represent the 95% confidence intervals). (**a**) The risk of LTE accident by helicopter model. (**b**) The risk of LTE accident by number of main rotor blades. (**c**) The risk of LTE accident by engine type.

New results are provided in Fig. 2, and they address questions that have not been resolved before. The most salient ones are the following. The risk of LTE accident varies by more than an order of magnitude across the different helicopter models (Fig. 2a). The most LTE prone is the Hughes 269, and it exhibits about twice the risk of such accidents as the second on the list, the Bell 206. The Bell 407 exhibits the lowest risk of LTE accident. The risk of LTE accidents in four-bladed helicopters is 8 times lower than this risk in two-bladed helicopters, and this difference is statistically significant (p < 0.05) (Fig. 2b). No statistically significant difference in the risk of LTE accidents exists between two-bladed and three-bladed helicopters. Lastly, the risk of LTE accidents in helicopters with a reciprocating engine is about twice as high as that in helicopters equipped with a turboshaft engine, and this difference is statistically significant (p < 0.05) (Fig. 2c).

### Deep learning model and multivariate results

Six different machine learning models were carefully trained and tuned to capture the dependence between the incidence proportion of LTE accidents and the 12 helicopter features noted previously. The models were the K-nearest neighbors (KNN)^19^, Support Vector Classification (SVC)^20^, Naïve Bayesian (NB)^21^, Random Forest Classification (RFC)^22^, Gaussian Process Classification (GPC)^23^, and Deep Neural Network (DNN). The DNN model significantly outperformed the others in training and testing, and it exhibited the highest accuracy and no overfitting (details in the “Methods” section). The results that follow are based on our DNN model of LTE accidents.

**Figure 3:**
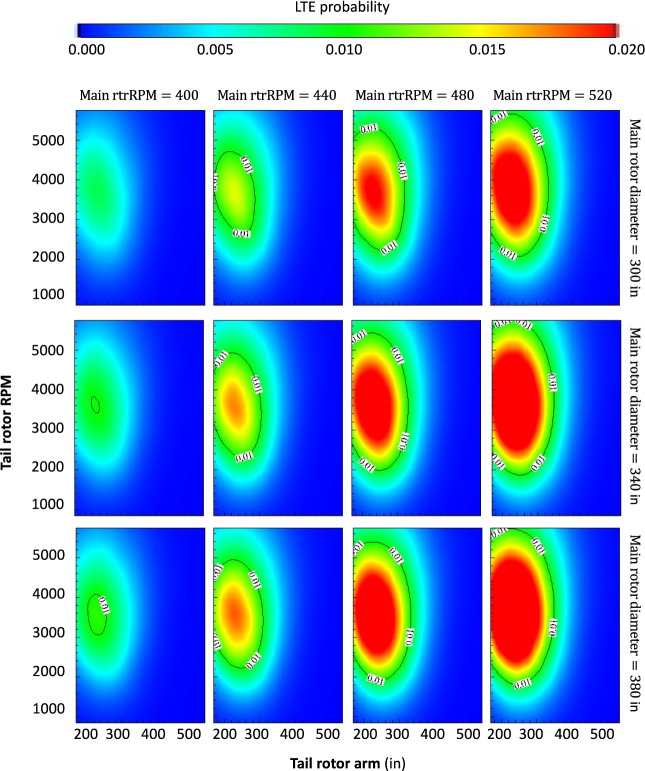
Multi-variate LTE risk analysis and results (helicopter with a reciprocating engine, 180 HP). The x- and y-axes in each panel are the tail rotor arm and tail rotor RPM respectively. The columns (left to right) stratify the results by increasingly higher main rotor RPM. The rows (top to bottom) stratify the results by increasingly larger main rotor diameter.

The results in Fig. 3 display the impact of four different variables on the risk or probability of LTE accident for a helicopter with a reciprocating engine and broadly similar features to an average Hughes 269.

Four initial variables in the helicopter design (features in our DNN model), the tail rotor arm and its RMP, and the main rotor diameter and its RPM, were identified as important drivers of the risk of LTE accidents. There are several key findings in this figure, the most salient are the following: (1) within each panel, a distinctive LTE danger zone exists in the feature space at the intersection of short tail rotor arm and high tail rotor RPM. We define an LTE danger zone as a sector in the feature space associated with a high probability of LTE accident (p > 0.01). Each of these features, along with their statistical interaction, contribute in a non-linear manner to the response variable. It can be stated that the combination of short tail rotor arm and high tail rotor RPM is associated with a higher risk of LTE; (2) moving rightward within each row, as the main rotor RPM increases, a clear increase in the LTE danger zone is observed. In other words, this third feature further modifies the response variable, and the risk of LTE increases as the main rotor RPM increases. It can thus be stated that a higher main rotor RPM is associated with a higher risk of LTE for the combination of short tail rotor arm and high tail rotor RPM; (3) moving downward within each column across different rows, as the main rotor diameter increases, a minor but discernible impact on the LTE danger zone is observed. In other words, this fourth feature further modifies the response variable, and the risk of LTE increases as the main rotor diameter increases, albeit to a lesser extent than with the previous feature. It can thus be stated that a larger main rotor diameter is associated with a slightly higher risk of LTE for the combination of short tail rotor arm and high tail rotor RPM.

The effects of the other features or helicopter design parameters on the risk of LTE were also carefully investigated. The results in Fig. 3 can be conceived of as a projection from a higher dimensional feature space (12) onto these matrix-like 2D contour plots. Consider, however, a third axis orthogonal to the figure and representing the engine horsepower (HP). We found that the LTE danger zone turns from the elliptical shapes in Fig. 3 to elliptical cylinders with the parameters of the ellipses exhibiting little to no variation along the new axis (from 180 to 360 HP). In other words, engine HP within this range appears to have little effect on the risk of LTE compared with the previous main effects identified in (1–3).

**Figure 4:**
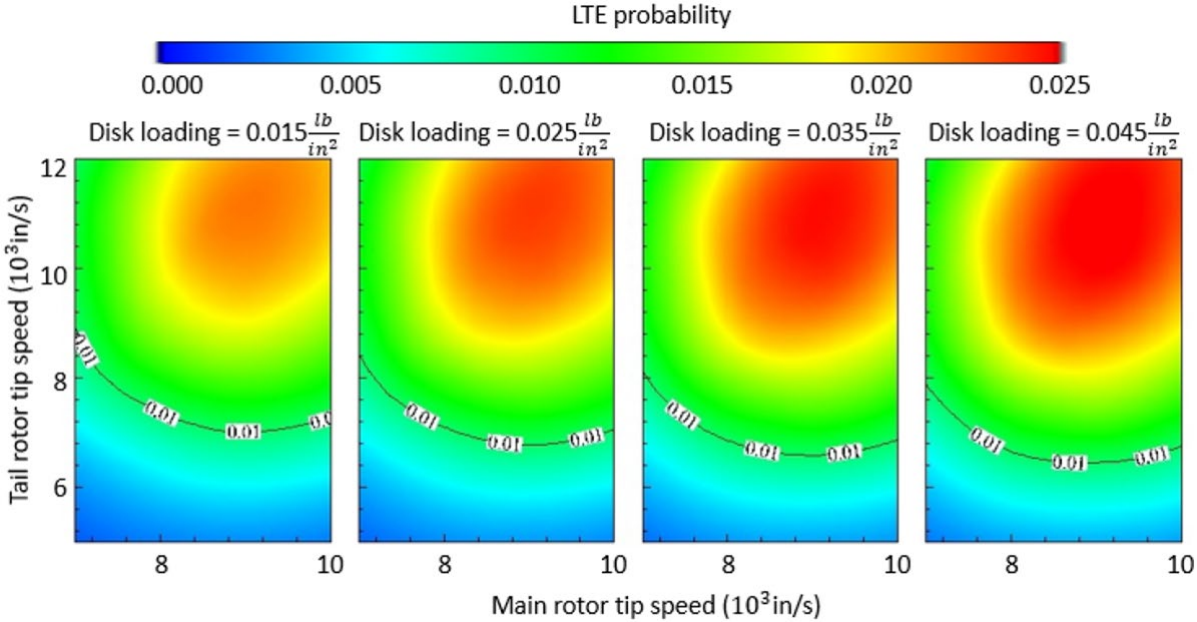
Multi-variate LTE risk analysis and results (helicopter with a reciprocating engine, 180 HP). The x- and y-axes in each panel are the main rotor tip speed and tail rotor tip speed respectively. The columns (left to right) stratify the results by increasingly higher disk loading (weight divided by disk area).

Following this analysis, we examined the risk of LTE as a function of “aggregate” design variables typically used in helicopter preliminary design, such as disk loading, main rotor tip speed, and tail rotor tip speed. We refer to these variables as aggregate since, for example, the main rotor tip speed is determined by the main rotor diameter and RPM, which are the parameters initially examined. The results are provided in Fig. 4.

Several findings are captured in these figures, the most salient are the following: (1) within each panel, the tip speeds of the main rotor and tail rotor contribute in a non-linear manner to the risk of LTE, and as a result, it can be stated that the combination of high main rotor tip speed and high tail rotor tip speed is associated with a higher risk of LTE; (2) moving rightward in Fig. 4, as the disk loading increases, the LTE danger zone clearly expands. In other words, this third parameter further modifies the response variable, and the risk of LTE increases as the disk loading increases. It can thus be stated that a high disk loading is associated with a higher risk of LTE for the combination of high main and tail rotor tip speeds. The tail rotor arm exerts the same effect on the risk of LTE as shown in Fig. 3 and is not repeated here for brevity.

**Figure 5:**
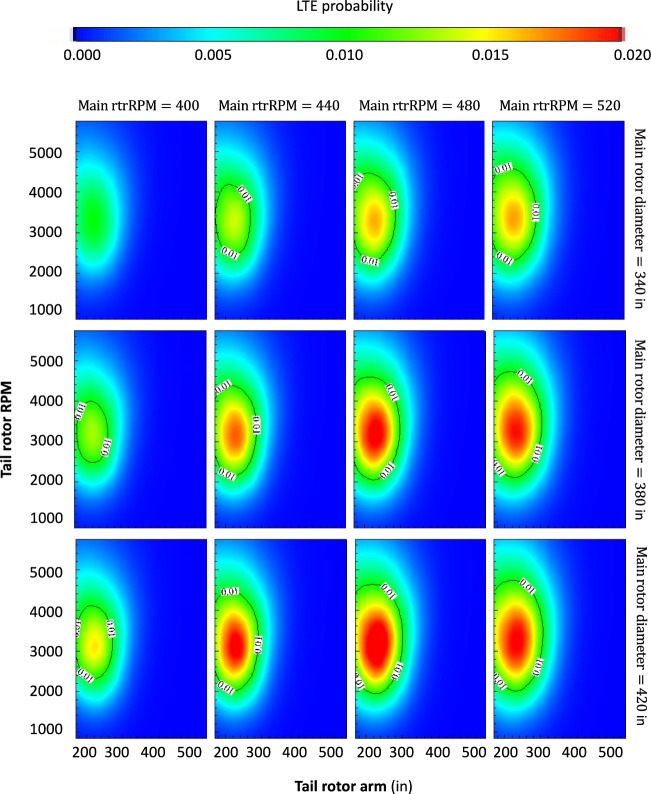
Multi-variate LTE risk analysis and results (helicopter with a turboshaft engine, 420 HP). The x- and y-axes in each panel are the tail rotor arm and tail rotor RPM respectively. The columns (left to right) stratify the results by increasingly higher main rotor RPM. The rows (top to bottom) stratify the results by increasingly larger main rotor diameter.

The effect of the engine type is examined next for a helicopter with a turboshaft engine and broadly similar features to an average Bell 206. The results are provided in Fig. 5.

**Figure 6:**
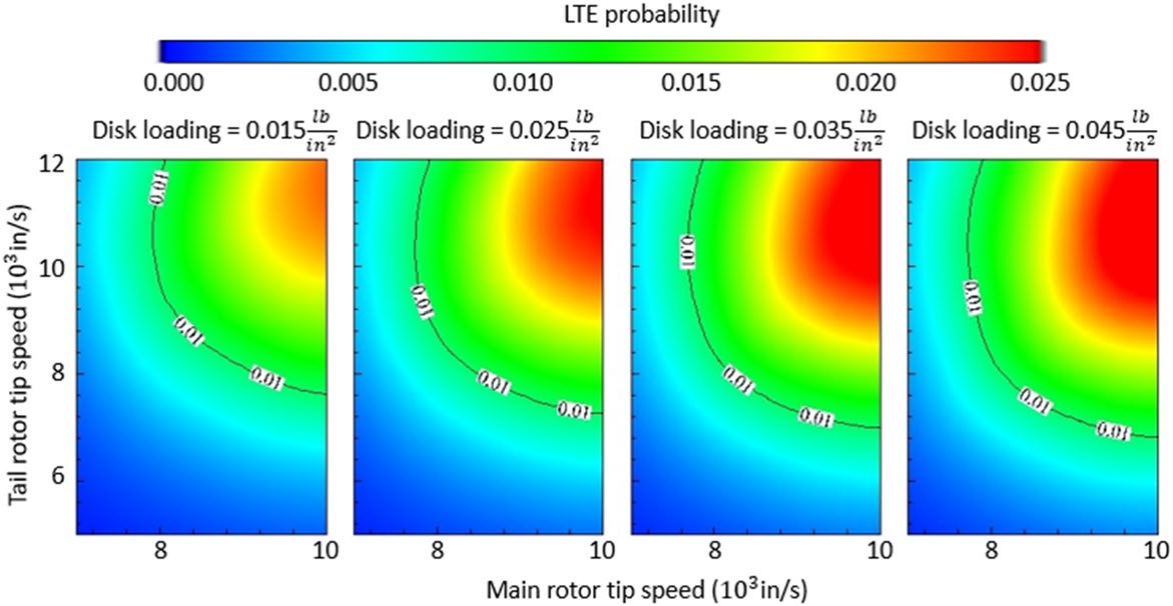
Multi-variate LTE risk analysis and results (helicopter with a turboshaft engine, 420 HP). The x- and y-axes in each panel are the main rotor tip speed and tail rotor tip speed respectively. The columns (left to right) stratify the results by increasingly higher disk loading (weight divided by disk area).

The same results as the previous ones are found in the case of helicopters with turboshaft engines. Namely, (1) a distinctive LTE danger zone exists in the feature space at the intersection of short tail rotor arm and high tail rotor RPM; (2) an increase in the risk of LTE as the main rotor RPM increases; (3) a more prominent increase in the risk of LTE as the main rotor diameter increases than for helicopters with reciprocating engines. This constitutes the main difference between the reciprocating and turboshaft cases. The results of the risk of LTE as a function of “aggregate” design variables are provided in Fig. 6.

The same interpretation of the results in Figs. 4 and 6 applies to the helicopters equipped with reciprocating and turboshaft engines. We note that both types exhibit similar variations, albeit more pronounced for the reciprocating ones, of the risk of LTE with these three parameters, disk loading, and main rotor and tail rotor tip speeds.

We were not able to examine the impact of the solidity factor on the risk of LTE because of the paucity of this data in the public domain.

It is important to acknowledge that these findings are *associations* between predictors and a response variable, and they should not be interpreted as causal in nature. The causal pathways, if it exists, may be mediated by other factors and cannot be uncovered with statistical analysis based on observational data, as done in this work. Nevertheless, clear associations between the risk of LTE and several helicopter design variables have been uncovered, and previously unknown danger zone in the feature space discovered. The association are physically and aerodynamically plausible. These findings can serve as a basis for further investigation, for example in computational fluid dynamics (CFD) of main rotor disk vortex interference with tail rotor, or tail rotor vortex ring state, or in new research directions to deepen our understanding of the physical mechanisms underpinning LTE accidents for better prevention.

## Discussion

It is frequently stated and generally accepted in the rotorcraft community that 60–70% of all helicopter accidents are due to pilot errors, including LTE accidents^16,24^. This narrative is strongly entrenched and is rarely, if ever, questioned. The absence of black boxes on the majority of helicopters—less than 10% are equipped with such devices^25^—makes it difficult to challenge this viewpoint.

This work challenges this myopic narrative in the context of LTE accidents. It demonstrates that an intrinsic propensity to these types of accidents is embedded in the helicopter design and configuration and is beyond merely pilot errors. For example, given the results provided in Fig. 2a, it is difficult to explain away the differences in risk of LTE of the R22 or Bell the 407, for example, compared with the Hughes 269 (3 and 15 times respectively lower risk of LTE in the two former helicopters than the latter). Not acknowledging this possibility of fundamental LTE risk factors embedded in the helicopter design and configuration, limits the scope of possible solutions for tackling this persistent problem.

If progress is to be made toward an end-goals of eliminating LTE accidents, it is important to ask new research questions about this phenomenon and to examine new venues for understanding and tackling it. Better training for pilots to handle LTEs is always warranted, but a more effective safety strategy is to design helicopters that either avoid this problem altogether in the first place, or that make it easier for pilots to get out of an LTE should they find themselves in such a situation.

This work first developed a scorecard for LTE accidents providing different descriptive statistics of these accidents. The LTE scorecard constitutes a useful first step toward a better understanding of these accidents, but the results are only based on NTSB (numerator) data and do not include measures of exposure to enable inferential and comparative analysis. In a second step, one measure of exposure was introduced and the incidence proportions, or risk of LTE, was derived for different types of helicopters. This enabled a comparative analysis and provided further insights into these accidents. For example, it was found the risk of LTE accidents in helicopters with reciprocating engines is about twice as high as that in helicopters equipped with turboshaft engines. The more important results were derived using a highly accurate DNN model, which captures the dependence between the incidence proportion of LTE accidents and 12 different helicopter features. The key finding is the existence of a danger zone in the feature space at the intersection of short tail rotor arm and high tail rotor RPM. Similarly, it was found that the combination of high main rotor tip speed and high tail rotor tip speed is associated with a higher risk of LTE, and that increasing the disk loading further expands the LTE danger zone.

This work should be considered in light of its limitation, the most patent is the possibility that the results are confounded by unaccounted for variables, in particular helicopter usage, which in the absence of flight-hour data, cannot be controlled for. Nevertheless, these results deserve careful attention from the rotorcraft community, especially helicopter manufacturers and regulators. How to exit this danger zone in the feature space, should further physics and simulation-based analysis such as CFD confirm it is indeed a causal factor in LTE accidents, is left as a fruitful venue for future work.

## Methods

### Deep neural network architecture

We developed a novel LTE probability prediction model with the tools of deep learning (DL) via a deep neural network (DNN). DL enables the learning of complex functions with multiple layers of transformation, starting with the input variables and proceeding to more abstract levels of representation^26^. We designed a DNN with six layers: an input layer with 12 input features, four hidden layers (the first three layers with Leaky-ReLU activation layers)^27^, and an output layer with a single output as the LTE probability. The hidden layers consist of 500, 1000, 500, and 1 neurons, respectively. We arrived at this network structure through careful analysis and experimentation with different layers and neuron sizes. The performance objective was to obtain the best accuracy while avoiding overfitting and underfitting, as explained in the next paragraph. We convert the fourth hidden layer, which consists of one single neuron, to the output of the LTE probability through a sigmoid activation function^28^. We used the dropout method and L2 regularization to avoid overfitting^29^, and we adopted the binary cross entropy^21^ (BCE) as the cost function with an ADAM^30^ optimizer in the backpropagation process.

**Figure 7:**
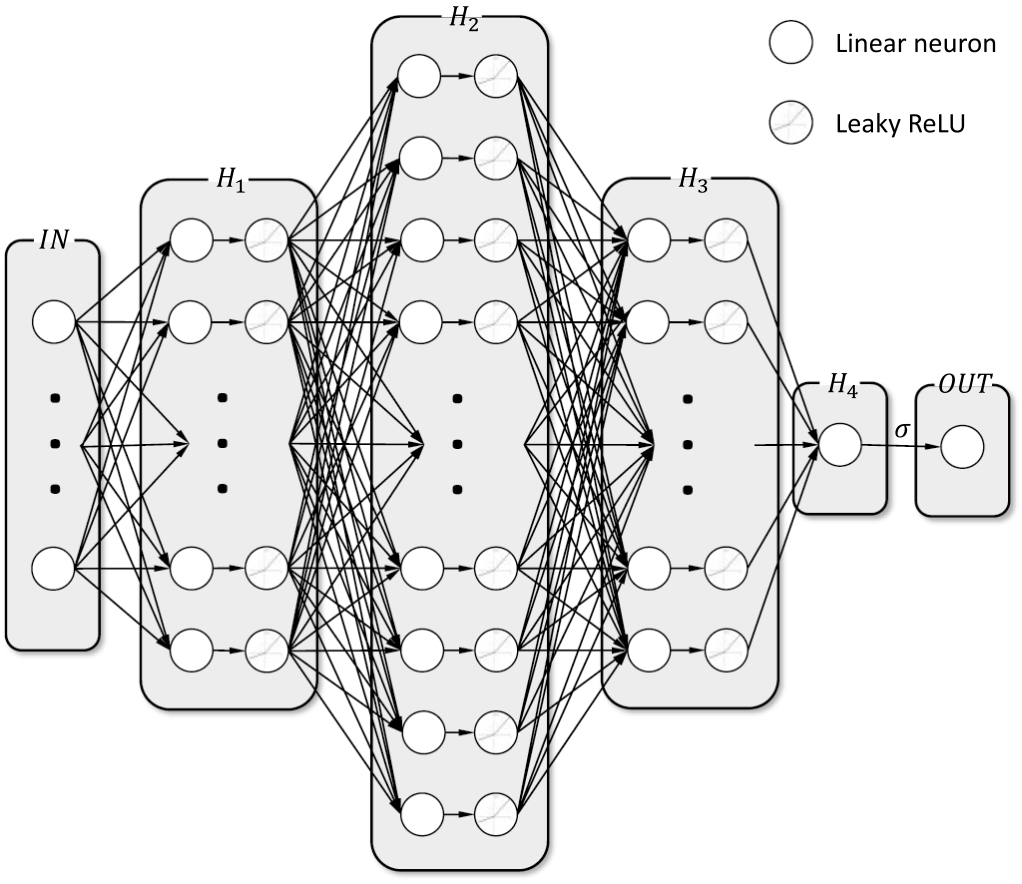
The DNN Architecture for the LTE probability prediction model (*H*_1_, *H*_2_, *H*_3_ and *H*_4_ refer to the first, second, and third hidden layer, respectively).

An important tradeoff arises in the design of the DNN architecture and the selection of its neuron distribution. On the one hand, increasing the number of neurons will improve the accuracy of the DNN for the training dataset. On the other hand, a DNN with too many neurons will likely suffer from overfitting, in which case the predictive capabilities on new datasets (generalization) would degrade. A good neuron distribution is one that achieves accurate performance on both the training and validation sets. We selected the DNN structure that simultaneously (1) minimized the performance gap on the training and validation sets and (2) achieved high accuracy on both sets (to avoid overfitting and underfitting). This was the 12–500–1000–500–1–1 neuron distribution noted previously with four hidden layers. Our final DNN model structure is depicted in Fig. 7.

### K-fold cross-validation

In designing and adjusting our DNN model architecture, we used the k-fold cross-validation technique to avoid overfitting and to assess how well the performance of the DNN will generalize to independent datasets not used in the training of the DNN^31,32^. In the k-fold cross-validation method, the dataset is randomly split into k-subgroups or k-folds. Iterating through each fold, we set aside one-fold as the “validation set” and the other k − 1 folds as the “training set”. During each of the k-iterations, we optimized (trained) the DNN parameters on the training set then assessed its performance on the validation set. We then modified the DNN architecture, retrained the model, and repeated this assessment process on the other validation sets. We chose k = 10, which is typically used in practice because it provides a good balance in the bias-variance tradeoff^33^.

### Comparison between DNN and alternative classifications

**Figure 8:**
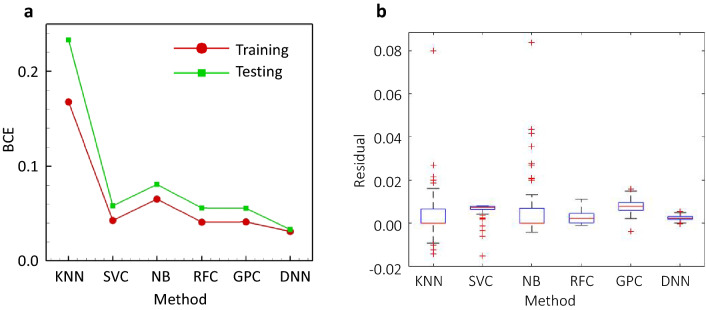
Performance comparison of the DNN and other shallow machine learning LTE models. (**a**) Binary Cross Entropy (BCE) for the training and testing of the different model: K-Nearest Neighbor (KNN)^19^, Support Vector Classification (SVC)^20^, Naïve Bayesian (NB)^21^, Random Forest Classification (RFC)^22^, and Gaussian Process Classification (GPC)^23^. (**b**) LTE prediction residuals of the different models.

To examine the effectiveness of our DNN model, we compare its performance with alternative shallow machine learning classification methods, including K-Nearest Neighbor (KNN), Support Vector Classification (SVC), Naïve Bayesian (NB), Random Forest Classification (RFC), and Gaussian Process Classification (GPC). We calculate the BCE error for both training and testing in the k-fold cross-validation for different classifications to evaluate the model accuracy, as shown in Fig. 8a, and we provide the prediction residuals for the six different models examined in Fig. 8b.

As shown in Fig. 8, the DNN model is the most accurate, and it exhibits the least bias and is free of overfitting.

## Supplementary Information

Supplementary Video 1.

Supplementary Video 2.

Supplementary Legends.

## Data Availability

The data in this work is publicly available on the National Transportation Safety Board (NTSB) (https://www.ntsb.gov/) and FAA (https://www.faa.gov/) websites. The websites allow the user to apply several filters before downloading the data.
