# Supplementary material for: Data-driven analysis and new findings on the loss of tail rotor effectiveness in helicopter accidents
Source: Sci Rep. 2022 Feb 16;12:2575. doi: 10.1038/s41598-022-06647-0 (PMC8850462; doi:10.1038/s41598-022-06647-0)
Supplement: Supplementary file 3 — Supplementary Legends. [file 41598_2022_6647_MOESM3_ESM.pdf]

Supplementary

Supplementary video 1: Loss of a tail rotor effectiveness of a Hughes 269 (Florida, USA, 2015)

Supplementary video 2: Loss of a tail rotor effectiveness of a Bell 206 (L'Aquilla, Italy, 2017)
